# Metabolomic Response of *Calotropis procera* Growing in the Desert to Changes in Water Availability

**DOI:** 10.1371/journal.pone.0087895

**Published:** 2014-02-10

**Authors:** Ahmed Ramadan, Jamal S. M. Sabir, Saleha Y. M. Alakilli, Ahmed M. Shokry, Nour O. Gadalla, Sherif Edris, Magdy A. Al-Kordy, Hassan S. Al-Zahrani, Fotouh M. El-Domyati, Ahmed Bahieldin, Neil R. Baker, Lothar Willmitzer, Susann Irgang

**Affiliations:** 1 Department of Biological Sciences, Faculty of Science, King Abdulaziz University (KAU), Jeddah, Saudi Arabia; 2 Agricultural Genetic Engineering Research Institute (AGERI), Agriculture Research Center (ARC), Giza, Egypt; 3 Genetics and Cytology Department, Genetic Engineering and Biotechnology Division, National Research Center, Dokki, Egypt; 4 Department of Genetics, Faculty of Agriculture, Ain Shams University, Cairo, Egypt; 5 Max-Planck-Institut für Molekulare Pflanzenphysiologie, Potsdam-Golm, Germany; 6 Department of Biological Sciences, University of Essex, Colchester, United Kingdom; Universidade Federal de Vicosa, Brazil

## Abstract

Water availability is a major limitation for agricultural productivity. Plants growing in severe arid climates such as deserts provide tools for studying plant growth and performance under extreme drought conditions. The perennial species *Calotropis procera* used in this study is a shrub growing in many arid areas which has an exceptional ability to adapt and be productive in severe arid conditions.

We describe the results of studying the metabolomic response of wild *C procera* plants growing in the desert to a one time water supply. Leaves of *C. procera* plants were taken at three time points before and 1 hour, 6 hours and 12 hours after watering and subjected to a metabolomics and lipidomics analysis. Analysis of the data reveals that within one hour after watering *C. procera* has already responded on the metabolic level to the sudden water availability as evidenced by major changes such as increased levels of most amino acids, a decrease in sucrose, raffinose and maltitol, a decrease in storage lipids (triacylglycerols) and an increase in membrane lipids including photosynthetic membranes. These changes still prevail at the 6 hour time point after watering however 12 hours after watering the metabolomics data are essentially indistinguishable from the prewatering state thus demonstrating not only a rapid response to water availability but also a rapid response to loss of water.

Taken together these data suggest that the ability of *C. procera* to survive under the very harsh drought conditions prevailing in the desert might be associated with its rapid adjustments to water availability and losses.

## Introduction

Drought is one of the most serious limitations for agriculture limiting plant growth, photosynthesis and, thus, productivity in many areas of this planet. Given the anticipated climate change, it is expected to worsen in the future thus becoming an even more important threat for global food supply [1]. Due to the extraordinary importance of drought stress, many studies have been performed aiming at an improved understanding of the mechanisms underlying drought tolerance with the hope to ultimately translate this knowledge into improved crop varieties.

Essentially, a variety of different approaches have been followed in different plant systems. Starting from the observation that response to drought/drought tolerance is a multigenic trait, quantitative genetic studies taking advantage of natural diversity with respect to drought tolerance have been performed. Many of these studies have been performed in crop species such as corn or rice [2], [3] and improved varieties, e.g. for corn have been developed [4], [5]. These studies have furthermore led to the identification of QTL's associated with improved performance under water limiting conditions and recently also to the molecular cloning of underlying genes such as DRO1 from rice [6].

As to the molecular responses, numerous studies have been performed in different plant systems such as *A. thaliana* or corn [7], [8], resurrection plants, e.g. *Craterostigma plantagineum*, displaying extreme drought/desiccation tolerance [9], [10] or following a sister group contrast approach by comparing two closely related species differing significantly in drought tolerance [11], [12]. These studies showed that responses to limiting water availability at the organismal and cellular levels include inhibition of growth, stomatal closure and reduced photosynthesis. Further responses include the accumulation of osmoprotectants such as sugars, sugar alcohols and amino acids, such as proline, and a change in the glutathione/ascorbate cycle to probably combat oxidative stress. These changes are accompanied by a plethora of further biochemical and gene expression changes aiming at keeping the negative consequences of the limited water availability at a minimum [2], [9], [13]–[19]

Membranes are very sensitive to dehydration and in consequence lipid composition changes to cope with this stress [20]. Major changes observed consistently as a result of dehydration are a decline in galactolipids, an increase in digalactosyldiacyglycerol (DGDG) as compared to monogalactosyldiacylglycerol (MGDG), a decline in the degree of fatty acid desaturation [19]–[21] and an increase in triacylglycerols (TAG) [19].

In this study we describe the metabolomic and lipidomic response of a wild plant species, *Calotropis procera*, growing in the desert near Jeddah in Saudi Arabia to sudden water availabity.

*Calotropis procera* belongs to the spurge family (*Euphorbiaceae*). This family includes about 280 genera and 8000 species which occur in tropical and in temperate regions all over the world. The genus *Calotropis* is distributed around sub-tropical and tropical regions of Asia and Africa and is used in traditional medicine.

In addition *Calotropis procera* displays an impressive drought tolerance given the fact that it grows in extremely dry areas such as the Saudi-Arabian desert thus making it an interesting model for studying the response to extreme drought respectively scarce water supply.

We here describe the metabolomic and lipidomic response of *C. procera* with respect to rehydration. To this end wild plants growing in the desert near to Jedda, Saudiarabia were sampled at different time intervals before and after giving a onetime water supply thus providing a first insight into the metabolic response under true field conditions. Even given that the metabolomic and lipidomic data presented are only semiquantitative the results obtained demonstrate that *C. procera* has the ability to respond very fast (within one hour) to changes in water availability by reorienting its metabolism. With equally impressive speed, the plant responds to the loss of water by transforming its metabolism back to pre-watered state. This fast response may be a crucial parameter to survive under the harsh conditions prevailing in the Saudiarabian desert.

## Materials and Methods

### Plant material, location of sampling and watering

The experiment was conducted using plants grown wild in the desert near to Jedda. No specific permission was required to perform these experiments as this area is used routinewise by the Colleagues from the King Abdullaziz University. The plant used in these experiments Calotropis procera is not an endangered or protected species.

**Figure 1:**
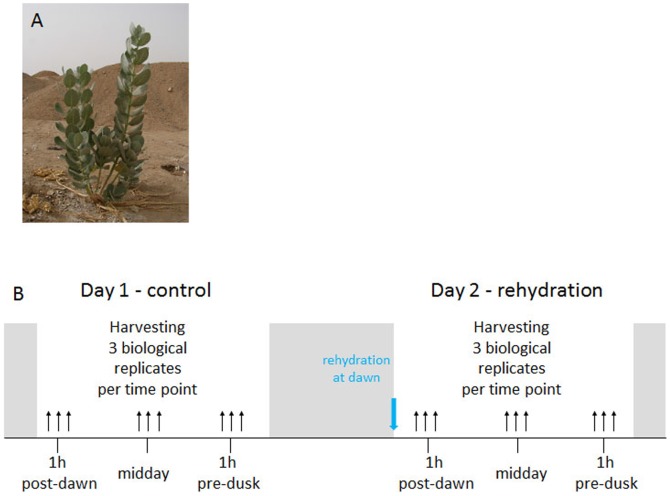
Experimental Setup and plants chosen. (a) Representative photo of the plants chosen for this experiment growing in its natural habitat in Saudi Arabia near to Jeddah. For this study representative species of similar size and performance were chosen. (b) Experimental set-up. At day 1 (control) leaves of three independent plants were harvested 1 h post-dawn, at midday and 1 h pre-dusk. One day later (Day 2), plants were watered at dawn and leaves were harvested 1 h post dawn, at midday and 1 h pre-dusk. Harvested leaves were frozen immediately in liquid –N and processes as described in Experimental procedures.

This experiment was conducted during September 2012 in the desert about 30 km away from Jeddah (latitude 21°26′6.00, longitude 39°28′3.00). The average temperature during the time the experiments were conducted varied from 28–37°C, humidity was 70–75%. In this area, *Calotropis procera* shrubs grow as single plants in the wild (cf. figure 1). For the conduction of this experiment, three *C. procera* plants of equal size were selected for watering (25 liters dH_2_O) and leaf sampling. Average rainfall in the Jeddah province in winter is ∼25 cm distributed over 10 individual days. Thus the the expected amount of water received by individual plant in one day in 1 m^2^ equals 25,000 cm^3^, i.e., 25 liters dH_2_O. Single similar-sized plants were given this amount of water in the evening. In the next day, we determined by visual inspection how far the water has penetrated. Water was no longer detectable 36 hours after watering as determined by a frequency domain probe CS615-FDR (Campbell Scientific Inc., Utah, USA). Plot edges were raised to avoid water flowing away and watering was done gradually over a period of 5 minutes to avoid spillage.

### Execution of the experimental treatment

#### a. Determination of relative water content (RWC)

Leaves of the three *C. procera* plants selected for this experiment were sampled one day before and three consecutive days after watering. Leaves at 50 cm from the ground were the targets for all samples. Leaf samples for RWC were immediately weighed and fresh weight (FW) determined. Leaves were transferred to sealed amber flasks, rehydrated in one L of water for five hours until fully turgid at 48°C, surface dried, and reweighed (turgid weight, TW). The leaf samples were then oven-dried at 72°C for 48 hours and reweighed (dry weight, DW) (Silva *et al*., 1996). RWC was calculated by the following formula:

RWC (%) equals (FW – DW) divided by ( TW – DW)×100.

Multiple comparisons were performed following the procedure outlined by Duncan's New Multiple Range test.

#### b. Metabolomics analysis

Samples for metabolomic studies were taken one hour (at dawn), six (at midday) and 12 hours (one hour pre-dusk) after water treatment. In order to be able to identify possible changes in metabolism due to diurnal fluctuations, samples were in addition taken one day before watering at the same three time points. Samples taken were frozen in liquid nitrogen and kept at −80 C until extraction. Three independent but comparable plants were used for this experiment, thus representing three biological replicates.

Leaf samples were extracted and processed for metabolomics analysis as detailed below [22], [23].

Approximately 100 mg of the frozen plant tissue was homogenized in 2-ml Eppendorf tubes twice for 1 min at maximum speed within a Retschmill. The metabolites were extracted from each aliquot in 1 ml of a homogenous mixture of −20°C methanol: methyl-tert-butyl-ether: water (1∶3∶1), with shaking for 30 min at 4°C, followed by another 10 min of incubation in an ice cooled ultrasonication bath. After adding 650 µl of UPLC-grade methanol: water 1∶3, the homogenate was vortexed and spun for 5 min at 4°C in a table-top centrifuge. The addition of methanol: water leads to a phase separation, providing the upper organic phase, containing the lipids, a lower aqueous phase, containing the polar and semipolar metabolites, and a pellet of starch and proteins at the bottom of Eppendorf tube. The separate phases are isolated and dried down in a speed vac and stored at −80°C until use in the different metabolomic or lipidomic analyses.

UPLC-FT-MS measurement of lipids and semipolar metabolites and GC-TOF analysis of primary metabolites

UPLC separation of the semipolar fraction of the fractionated metabolite extract is performed using a Waters Acquity UPLC system, using an HSS T3 C18 reversed-phase column (100 mm ×2.1 mm ×1.8 µm particles; Waters). The mobile phases are 0.1% formic acid in H2O (Buffer A, ULC MS grade; BioSolve, http://www.biosolve-chemicals.com) and 0.1% formic acid in acetonitrile (Buffer B, ULC MS grade; BioSolve). A 2-µl sample (the dried-down aqueous fraction was re-suspended in 100 µl of UPLC grade water) is loaded per injection, and the gradient, which is taken out with a flow rate of 400 µl min−1, is: 1 min 99% A, 13-min linear gradient from 99% A to 65% A, 14.5-min linear gradient from 65% A to 30% A, 15.5-min linear gradient from 30% A to 1% A, hold 1% A until 17 min, 17.5-min linear gradient from 1% A to 99% A, and re-equilibrate the column for 2.5 min (20-min total run time).

The lipid fraction of the fractionated metabolite extract is performed on the same UPLC system using a C8 reversed-phase column (100 mm ×2.1 mm ×1.7 µm particles; Waters). The mobile phases are water (UPLC MS grade; BioSolve) with 1% 1 M NH4Ac, 0.1% acetic acid (Buffer A,) and acetonitrile: isopropanol (7∶3, UPLC grade; BioSolve) containing 1% 1 M NH4Ac, 0.1% acetic acid (Buffer B). A 2-µl sample (the dried-down organic fraction was re-suspended in 500 µl of UPLC-grade acetonitrile: isopropanol 7∶3) is loaded per injection, and the gradient, which was taken out with a flow rate of 400 µl min−1, is: 1 min 45% A, 3-min linear gradient from 45% A to 35% A, 8-min linear gradient from 25% A to 11% A, 3-min linear gradient from 11% A to 1% A. After washing the column for 3 min with 1% A, the buffer is set back to 45% A and the column is re-equilibrated for 4 min (22-min total run time).

The mass spectra are acquired using an Exactive mass spectrometer. The spectra are recorded alternating between full-scan and all-ion fragmentation-scan modes, covering a mass range from 100 to 1500 m/z. The resolution is set to 10 000, with 10 scans per second, restricting the loading time to 100 ms. The capillary voltage is set to 3 kV with a sheath gas flow value of 60 and an auxiliary gas flow of 35 (values are in arbitrary units). The capillary temperature is set to 150°C, whereas the drying gas in the heated electrospray source is set to 350°C. The skimmer voltage is set to 25 V, whereas the tube lens is set to a value of 130 V. The spectra are recorded from 1 min to 17 min of the UPLC gradients.

The polar phase is analyzed for primary metabolites using an established GC-TOF ms protocol [22], [23].GC-TOF chromatograms were extracted and annotated as described by [24]. Lipid annotation was based on retention times, exact molecular mass and comparison to an inhouse database. For statistical analysis and visualization (ANOVA, Bonferroni correction, PCA, boxplots) the R-software was used (http://cran.r-project.org/). ANOVAs were conducted using the harvesting time and condition (control and watering) as factors and resulting p-values were corrected for multiple testing using the stringent Bonferroni method. For principal component analysis (PCA), the “bpca”- algorithm of the “pcaMethod”- package was used [25]. Heatmaps were visualized using the Multi experiment Viewer software (MeV) version 4.8.1.

## Results and Discussion

### Experimental set-up

In a preliminary experiment leaf samples were taken from three independent plants of similar stature and developmental stage (cf. Figure 1 for a representative plant) at the end of the day (one hour pre-dusk), at seven days and one day before watering; and two and seven days after watering and subjected to our metabolomics platforms. An ANOVA analysis of the metabolomic data showed that the watering had no influence on the metabolite composition (data not shown).

The absence of a significant effect of watering on the metabolism of the treated plants could have two explanations: either metabolism of *Calotropis procera* is highly buffered and does not respond to water treatment or the effect on metabolism is much more transient and already lost two days after water treatment. To distinguish between these possibilities, we devised a second experiment where we collected samples within a more narrow time window, i.e., one (at dawn), six (at midday) and 12 hours (one hour pre-dusk) after water treatment. In order to be able to identify possible changes in metabolism due to diurnal fluctuations, samples were taken one day before watering at the same three time points. Three independent but comparable plants were used for this experiment, thus representing three biological replicates. Samples were, again, immediately frozen and processed for analysis on our three metabolomics platforms (cf. Table S1 and Table S2 for all metabolomics data).

### Changes in primary, secondary and lipid metabolite contents of *Calotropis* plants due to watering

In order to see whether changes in metabolism are detectable at the earlier time-points, metabolite data from samples harvested within 12 hours after watering and obtained at the corresponding time points before watering were subjected to a principal component analysis.

**Figure 2:**
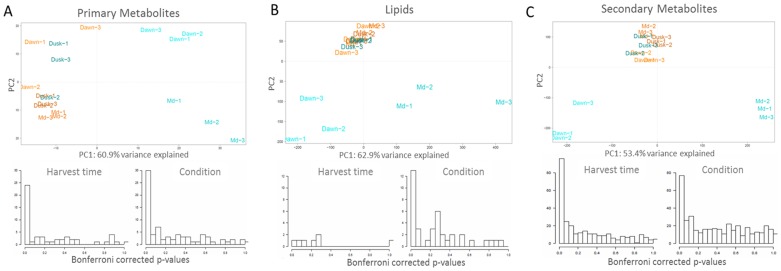
Principal Component Analysis (PCA) and ANOVA for metabolomic analysis of leaf samples from control and watered plants. (a) PCA (upper part) and ANOVA (lower part) for primary metabolites. (b) PCA (upper part) and ANOVA (lower part) for complex lipids. (c) PCA (upper part) and ANOVA (lower part) for secondary metabolites. Shown are always three independent samples per time point (dawn (1 hour post dawn/after watering), midday (6 hours after dawn/after watering) and pre-dusk (12 hours after dawn/after watering). Watered samples are shown in blue, non-watered in red. The lower part shows the results of a Bonferroni corrected ANOVA displaying the influence of treatment (watering) for all samples and of harvesting time for primary and secondary metabolites.

The results are shown in Figure 2, a–c revealing several interesting features:

There is a very clear and significant effect of watering on metabolism detectable on the level of primary, secondary and lipid metabolite content.
This effect is of a highly transitory nature. Thus, samples taken one and six hours after watering clearly differ from the non-treated samples, on one hand, and from each other, on the other hand. Besides, the effect of watering on metabolism vanishes after 12 hours (sample: at pre-dusk), thus confirming the results from the pilot experiment.
Some metabolites measured by the GC-MS platform (primary metabolism) vary with sampling time with the samples taken at dawn being different from those taken at the pre-dusk and midday. This was also evidenced by ANOVA, whereas no such influence is seen for the lipids (Figure 2d).
Figure 2a–c, furthermore, indicates a high reproducibility of the experiment as evidenced by the clustering of the control samples and the “return” of the samples 12 hours after water treatment to the control level. This is remarkable given the fact that the entire experiment was performed under field conditions in the desert with individual plants grown and rainfed in the wild.

### Changes in amino acids, TCA cycle intermediates and sugar alcohols

**Figure 3:**
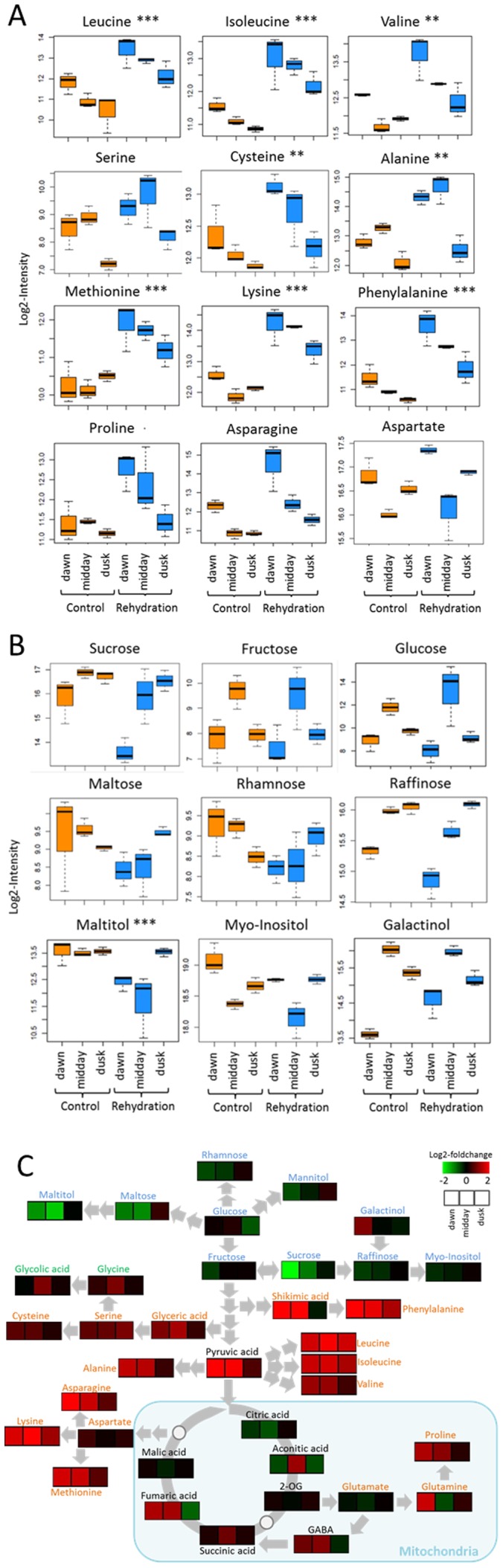
Boxplots and pathway visualization of representative primary metabolites. (**a**) and (b): Boxplot-visualizations for a subset of amino acids (A) and sugars and sugar alcohols(B) as determined for the three independent samples for the different time points and treatments as indicated on the x-axis. (b) Pathway mapping of a number of primary metabolites visualized as their averaged log2-foldchange ratio of rehydration versus control (green  =  decrease; red  =  increase).

**Figure 4:**
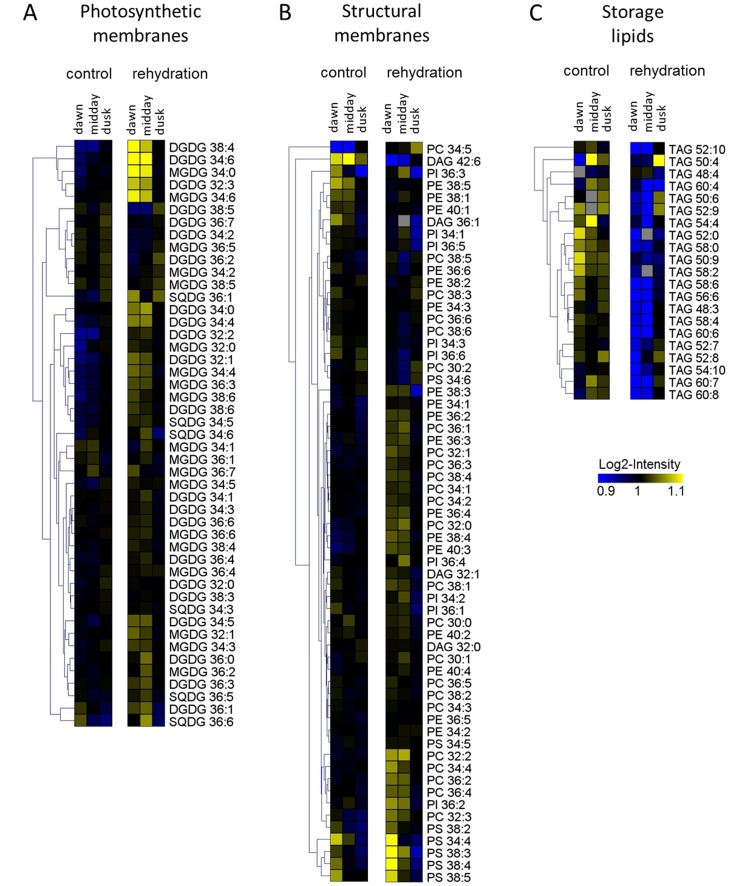
Clustered heatmap visualization of different lipid classes. Shown is the average abundance of several complex lipids visualized in a false-color heatmap at the three time points before and after watering ordered according to their presence in photosynthetic membranes, in cellular membranes or representing storage lipids.

In total, 357 primary metabolites could be detected via GC-TofMS analysis of which 118 could be annotated (cf. Table S1). Significant changes were revealed by ANOVA and PCA (figure 2). It should be mentioned that due to the fact that the relative water content increased in response to watering (cf. below) the relative metabolite content does change as well. However this effect is marginal (less than 10%) and the changes observed were as a rule much higher. To identify metabolites that force the separation of samples due to watering within the PCA, we used PCA-loading scores for each metabolite (cf. Table S1). Analysis of the main metabolites driving the separation in the PCA, respectively, the results of ANOVA of primary metabolites (Figure 2) reveal significant changes in amino acids, TCA cycle intermediates, sugars and sugar alcohols. Figure 3a–c presents box-plots of a number of typical examples and the pathways view is shown in Figure 4. The clearest trend is observed in behavior of the majority of amino acids. Thus, most amino acids respond to watering by a fast and significant increase in steady-state concentration. This effect is very pronounced for the branched-chain amino acids such as leucine, isoleucine or valine, in addition to phenylalanine, lysine, methionine, proline and asparagine. Notably, glutamine is not vastly exceeding the levels found in non-watered plants, whereas glutamic acid rather does not change or at midday is even lower as compared to the non-watered control. The reason for the observed increase in amino acids must remain unclear. One possible explanation would be an increased demand due to increased protein synthesis, another (opposite) explanation would be the degradation of (storage) proteins.

The increase in concentration for most amino acids seen here in case of *C. procera* as a result of watering is in contrast to most other metabolomics studies where amino acids were observed to increase in parallel to applying drought stress [8], [11], [12] Also in a more recent study using the resurrection lycophyte *S. lepidophylla*, more than half of the amino acids were more abundant in the dry as compared to the hydrated state [18].

We do not know the reason underlying these differences however except the use of a different plant system the experiments described here were performed under natural conditions in the field and furthermore the time intervals at which samples were taken after drought respectively rehydration treatment were as a rule longer in these studies as compared to our study. As described above all changes are of a highly transient nature.

Another aspect of our study worth mentioning is that the pattern of the amino acid concentration with respect to the three time points is very similar in the control and the watered conditions, i.e., the highest level is reached 1 hour after dawn in both control and watered samples with the concentrations subsequently decreasing towards midday and pre-dusk. Specifically the fact that the amino acid abundance in the samples taken at dawn before watering is also higher as compared to the midday and dusk samples is an independent confirmation of the response of the amino acids to watering.

Concerning TCA cycle intermediates the situation is similar though less pronounced. Whereas pyruvate and fumarate and, to a lesser extent, succinate display a significant increase as a result of watering, oxo-glutaric and malic acids essentially remain unchanged, whereas citric acid actually shows a decrease (cf. Table S1 and Figure 4). An increase in pyruvate and succinate has also been observed during rehydration of *S. lepidophylla* [18].

With respect to sugars and sugar alcohols, a more complex picture emerges. Glucose and fructose largely remain unchanged with only the one hour value being lower in the watered as compared to the non-watered control. Maltose is initially reduced in the water control which could be due to either an increase in maltose consumption or a decreased starch degradation. Sucrose, raffinose and maltitol are believed to serve as osmoprotectant. All three compounds display a significant reduction in watered as compared to non-watered control at the first two time points. This observation could be taken as indication that *C. procera*, senses during these early time points after watering as a relief from drought and, thus, osmotic stress and in consequence reduces the amount of compatible solutes. The members of the raffinose pathway, myoinositol and galactinol decrease transiently. As described above, proline like most other amino acids increases after rehydration. Proline is an accepted osmolyte and thus would be expected to decrease in parallel with the sugars and sugar alcohols. The increase observed could be either due to an increased need of proline for processes such as increased protein biosynthesis or due to a blockage of proline-consuming processes.

With respect to osmoprotecant sugars and sugar alcohols most studies analyzed their behavior in response to drought stress and not surprisingly an increase has been described in most studies [8], [11], [12], [18]. It should be noted however that in case of *S. lepidophylla*, some sugar alcohols were observed to increase after rehydration [18].

The significant transitory decrease in malonic acid described in our study is interesting when connecting this observation with the lipidomics data where we observed a transitory decrease in storage lipids (triacylglycerides). Taken together, this might suggest a reduced flux into storage lipids as an early response to watering.

Finally, it is noteworthy to comment on the behavior of glycolate and glycine (Figure 4 and Table S1). Glycine, in contrast to most other amino acids, reached its highest level at midday both in the watered and the non-watered control though the level is much higher in the watered sample. As we see the same pattern for glycolate, one possible though speculative explanation is that this increase is due to increased photorespiration which would also be in agreement with kinetics of serine accumulation (Figure 4a).

### Storage and membrane lipids

The non-polar phase of the extracts was subjected to UPLC-MS measurements and we identified 133 lipids belonging eight different classes: Diacylglycerol (DAG), Mono-galactosyl-diacylglycerol (MGDG), Di-galactosyl-diacylglycerol (DGDG), Sulfoquinovosyl-diacylglycerol (SQDG), Phosphatidylcholine (PC), Phosphatidylserine (PS), Phosphatidylinositol (PI), Phsophatidylethanolamine (PE), Triacylglycerols TAG). Lipid species within each class are characterized by the number of C-atoms and by the number of double bonds in the acyl-chains.

**Figure 5:**
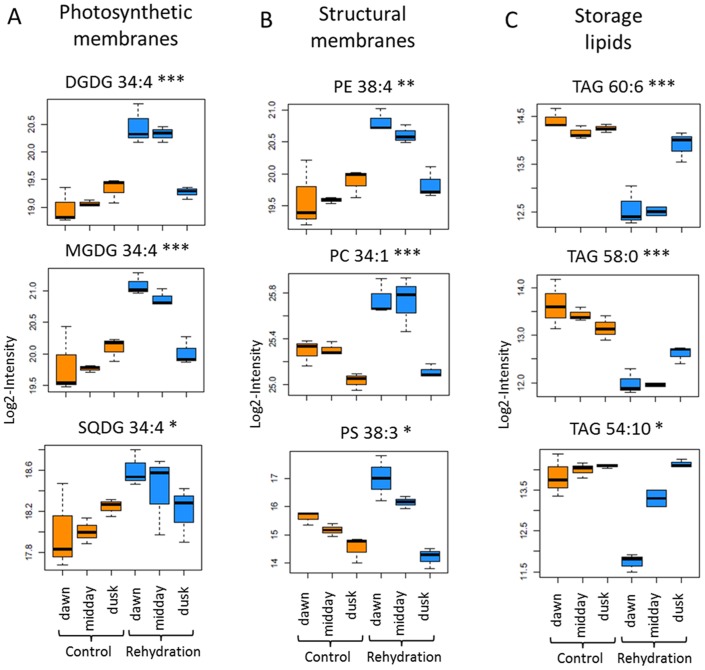
Boxplots of representative species of photosynthetic, structural and storage lipids. Boxplot-visualizations for a subset of complex lipids as determined for the three independent samples for the different time points and treatments as indicated on the x-axis of three replicates that were harvested at 1 h post-dawn, midday and 1 h pre-dusk for control and rehydrated plants.

As visible from Figure 2b, watering has a strong influence on complex lipid composition of *Calotropis* plants. A more detailed analysis shows that membrane lipids, in general and more specifically lipids of the photosystem, increase after watering. Most prominent examples comprise all MGDG's and the vast majority of the DGDG's (cf. Figure 5 a–d). A similar picture is observed for the majority of the SQDG's.

A contrasting picture emerges for the storage lipids, namely TAG's. Here for all classes, we observe a fast and significant decrease for the first two time points after watering and a reversion to the non-watered condition at the third time point.

These results largely agree with data described for other plant systems. Thus galactolipids have been described consistently to be reduced as a result of dehydration (thus mirroring the decline after the first transient increase after watering) although a change in the ratio between MGDG's and DGDG's is not obvious in our case. Also the transient decrease observed for TAG's as a result of watering is in agreement with the described data (an increase in TAG's as a result of drought stress;[19]). With respect to membrane lipids specifically phospholipids however the data are only in partial agreement with data reported for other systems [18], [19] which again might be contributed to the different plant system and/or the different experimental set-up.

### Secondary metabolites

Extracts of *Calotropis* are well known to display numerous pharmaceutical activities. Thus next to GC-MS measurements for primary metabolites, the polar phase was also subjected to secondary metabolite measurements by UPLC-MS. However, the secondary metabolism of *Calotropis* is only scarcely defined, thus, making annotation of compounds difficult. In order to have a first look into changes occurring on the secondary metabolite level, we identified m/z features, which is based on their exact mass and retention time behavior could putatively be assigned to some of the described secondary metabolites of *Calotropis*. No clear trend can be observed for secondary metabolites analyzed some of them increasing, others decreasing. The most significant increase amongst the putatively annotated compounds is observed for Uscharin, a compound with strong molluiscidal effects found in the latex of *Calotropis procera* (data not shown).

### Changes in relative water content (RWC) before and after watering

**Figure 6:**
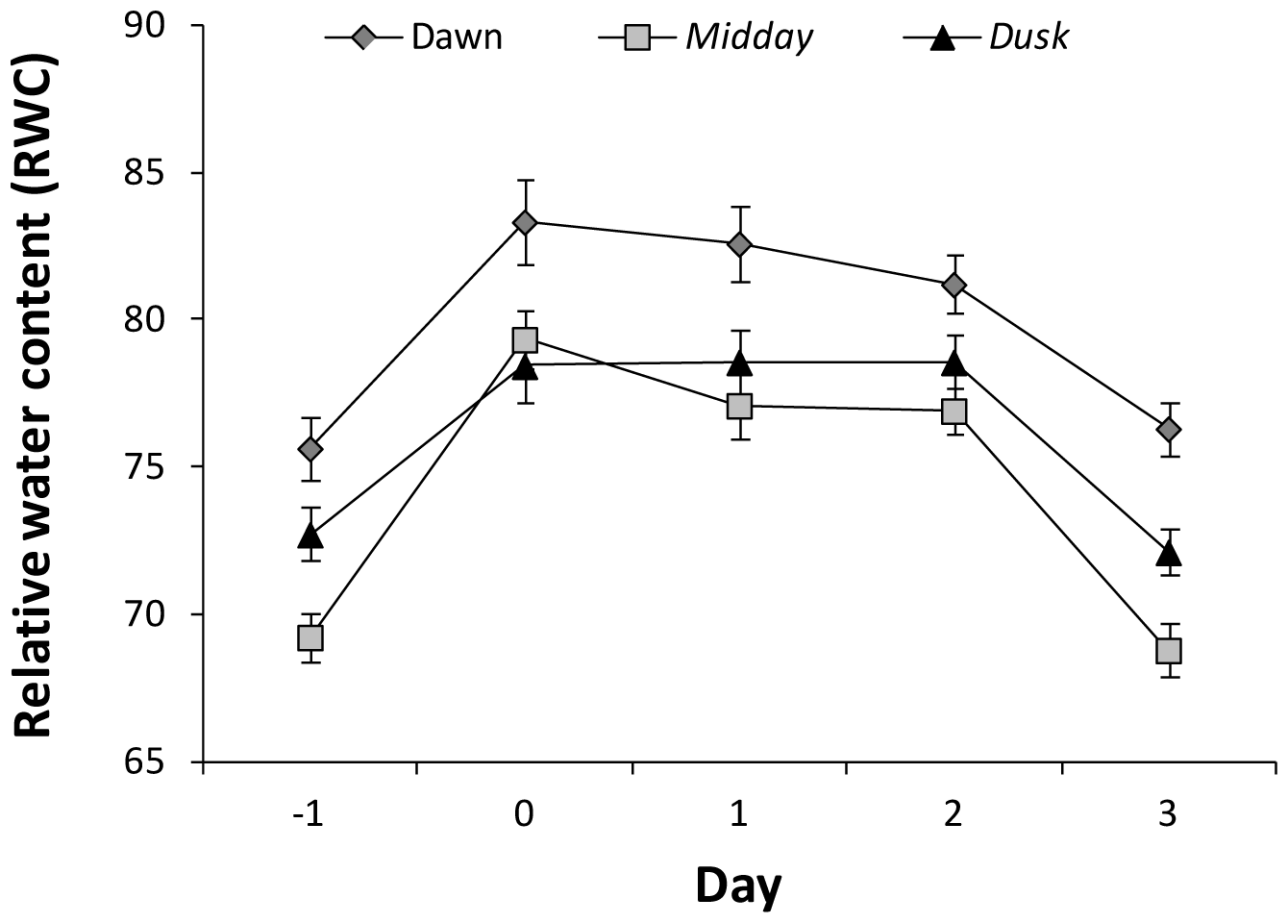
Relative water content of leaves of *C. procera* plants taken at dawn, midday and one hour pre-dusk one day before watering and up to three days after watering.

As shown in Figure 6, relative water content at dawn was shown to increase from 75% one day before watering to 83% one hour after watering, withheld for two more days before it started to fall back (76%) to control (before watering) levels at day three after watering. The same trend of results was reached at midday and pre-dusk, as RWC increased from 69% and 72%, respectively, one day before watering to 79% and 78%, respectively, in the day of watering, then fell back to pre-watering control level three days after watering. It is obvious that RWC was lowest at midday and highest at dawn across the five days, except at the day of watering, where RWC was higher at midday than at pre-dusk. The overall results indicate that *Calotropis* plant was able to hold water efficiently for two days, then returned to its original level of RWC prior to watering.

As evident from Figure 6 metabolism responds parallel to changes in RWC with respect to induction however the reversion of metabolism back to prewatering conditions is seemingly disconnected from the RWC which goes back to prewatering conditions much later, i.e. 3 days after watering. We are not in a position to explain this puzzling observation however one speculation is that metabolism at least in case of Calotropis does not directly respond to RWC as measured in the leaves but to other obviously still nonidentified parameters. One possibility could be water uptake as measured in the root system however as said this is first a pure speculation and second difficult to prove/disprove. Thus we are at present left with the description of this phenomenon.

## Conclusion

A time-resolved metabolomics and lipidomics response of *Calotropis procera*, a shrub growing in arid regions, towards the sudden supply of a limited amount of water is described. To increase the relevance of this study, the entire experiment was performed in the field respectively desert using wild grown *Calotropis* plants as experimental object.

Key observations are the transitory decrease in maltitol and raffinose (indicating a reduced drought stress), an increase in essentially all amino acids which might suggest that increased protein biosynthesis takes place, an increase in all structural lipids of the photosynthetic membranes (DGDG's, MGDG's, SQDG's) which may suggest that the plant prepares itself for increasing its photosynthetic capacity as well as an increase in most other membrane lipids.

Thus most changes observed and specifically their kinetics suggest that water availability in the natural habitat of *C. procera*, i.e. the desert, is such a scarce event that it has developed the capacity to respond fast and massively by remodeling it metabolic machinery towards growth. Understanding the molecular mechanisms behind this response may open new approaches for adapting crop plants to arid conditions.

## Supporting Information

Table S1 GC measured metabolite list and ANOVA values for individual metabolites.
(XLSX)

Table S2 Lipid list and ANOVA values for individual lipids.
(XLSX)
